# NUPACK: Computational Nucleic Acid Analysis and Design

**DOI:** 10.1021/acssynbio.5c00817

**Published:** 2026-04-02

**Authors:** Mark E. Fornace, Jining Huang, Cody T. Newman, Avinash Nanjundiah, Nicholas J. Porubsky, Marshall B. Pierce, Niles A. Pierce

**Affiliations:** † Division of Biology & Biological Engineering, 124492 California Institute of Technology, Pasadena, California 91125, United States; ‡ Division of Chemistry & Chemical Engineering, 6469 California Institute of Technology, Pasadena, California 91125, United States; § Applied Mathematics & Computational Research Division, 1666 Lawrence Berkeley National Laboratory, Berkeley, California 94720, United States; ∥ Division of Engineering & Applied Science, California Institute of Technology, Pasadena, California 91125, United States; ⊥ Consultant to the Division of Biology & Biological Engineering, 124492 California Institute of Technology, Pasadena, California 91125, United States

**Keywords:** DNA, RNA, 2′OMe-RNA, secondary structure, base-pairing, hybridization, complex ensemble, test tube ensemble, multitube ensemble, mixed-materials, equilibrium, partition function, concentration, ensemble defect, analysis, design, reaction pathway engineering, structural engineering including pseudoknots

## Abstract

NUPACK is a growing software suite for the analysis and design of nucleic acid structures, devices, and systems serving the needs of researchers in the fields of nucleic acid nanotechnology, molecular programming, synthetic biology, and across the life sciences. NUPACK algorithms have pioneered the treatment of complex and test tube ensembles containing arbitrary numbers of interacting strand species, providing crucial tools for capturing concentration effects essential to analyzing and designing the intermolecular interactions that are a hallmark of these fields. Analysis and design of multitube ensembles enable reaction pathway engineering of dynamic hybridization cascades and structural engineering including the possibility of pseudoknots. The all-new NUPACK 4 scientific code base offers enhanced physical models (coaxial and dangle stacking subensembles), dramatic speedups (20–120× for test tube analysis), increased scalability for large complexes (e.g., 30,000 nt), mixed materials (specified at nucleotide resolution), and diverse hard and soft sequence constraints for design. The all-new NUPACK web app (nupack.org) facilitates rapid job submission and result inspection with NUPACK 4 algorithms running in parallel on a hybrid cloud compute cluster that scales dynamically in response to user demand. NUPACK 4 algorithms can also be run locally using the all-new NUPACK Python module.

## Graphical abstract

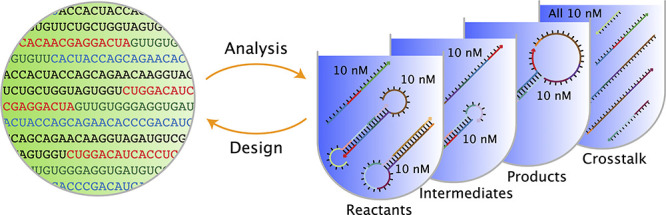

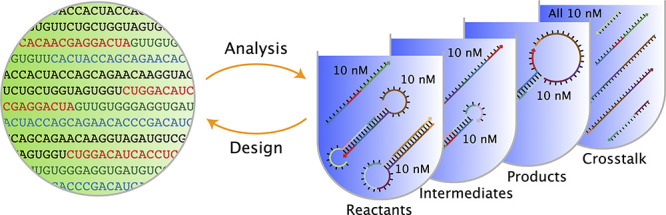

## Introduction

We are engaged in a multidecade effort to develop NUPACK (Nucleic Acid Package), a growing software suite for the analysis and design of nucleic acid structures, devices, and systems.^1^ NUPACK algorithms^2–10^ are formulated in terms of nucleic acid secondary structure (i.e., the base-pairs of a set of nucleic acid strands) based on empirical free energy models that have great utility for analyzing and designing functional nucleic acid systems. Mixed-material calculations are supported (RNA/DNA or RNA/2′OMe-RNA) with the material specified at nucleotide resolution.^10^

### Problems and Ensembles

NUPACK algorithms address two fundamental classes of problems:

• *Sequence analysis*: given a set of nucleic acid strands, analyze the base-pairing properties over a specified ensemble.
• *Sequence design*: given a set of desired base-pairing properties, design the sequences of a set of nucleic acid strands over a specified ensemble subject to diverse user-specified sequence constraints.

NUPACK algorithms operate over two fundamental ensembles:

• *Complex ensemble*: the ensemble of all (unpseudoknotted connected) secondary structures for an arbitrary number of interacting nucleic acid strands.
• *Test tube ensemble*: the ensemble of a dilute solution containing an arbitrary number of nucleic acid strand species (introduced at user-specified concentrations) interacting to form an arbitrary number of complex species.

NUPACK formulates and solves each of the four fundamental equilibrium problems defined on these ensembles: (1) equilibrium complex analysis;^5^ (2) equilibrium complex design;^6^ (3) equilibrium test tube analysis;^5^ and (4) equilibrium test tube design.^7^ Furthermore, to enable reaction pathway engineering of dynamic hybridization cascades, NUPACK formulates and solves: (5) kinetic test tube design^8^ using a multitube ensemble. Multitube ensembles also enable structural engineering including the possibility of pseudoknots.

### The Importance of Concentration Information

Note that a complex ensemble is subsidiary to a test tube ensemble, so complex analysis is inherent in test tube analysis (but not vice versa), and complex design is inherent in test tube design (but not vice versa). As it is typically infeasible to experimentally study a single complex in isolation, we recommend analyzing and designing nucleic acid strands in a test tube ensemble that contains the complex of interest as well as other competing complexes that might form in solution. For example, if one is experimentally studying strands A and B that are intended to predominantly form a secondary structure within the ensemble of complex A·B, one should not presuppose that the strands do indeed form A·B and simply analyze or design the base-pairing properties of that complex. Instead, it is more physically relevant to analyze or design a test tube ensemble containing strands A and B interacting to form multiple complex species (e.g., A, B, A·A, A·B, B·B) so as to capture both concentration information (how much A·B forms?) and structural information (what are the base-pairing properties of A·B when it does form?).

### All-New NUPACK 4 Scientific Code Base

NUPACK 4 analysis algorithms employ a new unified dynamic programming framework^9^ that provides enhanced secondary structure models (coaxial and dangle stacking subensembles), dramatic speedups (e.g., 20–120× for analysis of test tube ensembles), enhanced scalability for large complexes (e.g., an arbitrary number of strands comprising a total of 30,000 nt, and mixed materials (specified at nucleotide resolution). NUPACK 4 design algorithms leverage these performance benefits and support both hard constraints (that must be obeyed by the designed sequences; e.g., diversity and biological sequence constraints) and soft constraints (that penalize but do not prohibit suboptimal sequences; e.g., sequence symmetry and energy match constraints) for multitube ensembles.

### All-New Cloud-Based NUPACK Web App

Following its launch in 2007, usage of the NUPACK web app (nupack.org)^1^ increased to the point where the underlying static compute cluster was frequently overwhelmed by user demand. To provide a scalable resource for the worldwide research community, we rearchitected the NUPACK web app from the ground up to exploit a hybrid cloud compute cluster that scales dynamically in response to user demand. The all-new NUPACK web app integrates diverse components to create an intuitive and powerful analysis and design environment:

• *Algorithms*: mathematically rigorous, physically sound, computationally efficient scientific algorithms.^2–10^

• *Hardware*: a hybrid cloud compute cluster combining local hardware and scalable cloud hardware.
• *Interface*: an intuitive web interface for rapid job submission and result inspection.
• *Graphics*: publication-quality client-side graphics to enable straightforward interpretation of results, interactive data, and efficient preparation of talks and papers.

Researchers can run jobs and inspect results within the NUPACK web app, which leverages the NUPACK 4 scientific code base as its backend.

### All-New NUPACK Python Module

Alternatively, researchers can script and run jobs locally using the all-new NUPACK 4 Python module, providing the flexibility to interact with the broader Python ecosystem.

### Outline

To provide a foundation for describing the NUPACK web app, we begin by defining the physical model, relevant physical quantities, and the design formulation. For the convenience of the reader, definitions and descriptions are drawn from the original algorithms papers,^2–10^ but are here organized into a single unified presentation for easy perusal. We then summarize the features of the Analysis, Design, and Utilities pages of the NUPACK web app:

• *Analysis page*: Analyze the equilibrium base-pairing properties of one or more test tube ensembles (and subsidiary complex ensembles). These are the all-purpose sequence analysis tools.
• *Design page*: Design the sequences for one or more test tube ensembles (and subsidiary complex ensembles). These are the all-purpose sequence design tools.
• *Utilities page*: Analyze, design, or prepare figures for a single complex ensemble. These are quick tools applicable when your ensemble is a single complex.

## Physical Model

### Sequence

The *sequence*, ϕ, of one or more interacting strands is specified as a list of nucleotides ϕ^*a*^ for *a* = 1, ..., |ϕ|, where ϕ^*a*^ specifies both the material and base.

• For RNA nucleotides: ϕ^*a*^ ∈ {rA, rC, rG, rU}.
• For DNA nucleotides: ϕ^*a*^ ∈ {dA, dC, dG, dT}.
• For 2′OMe-RNA nucleotides: ϕ^*a*^ ∈ {mA, mC, mG, mU}.

Hence, for a mixed-material system (RNA/DNA or RNA/2′OMe-RNA), the sequence alphabet contains eight letters rather than four. Sequences are specified 5′ to 3′. The material need only be specified when there is a change in material (e.g., rACGdAT denotes a strand with 3 RNA nucleotides followed by 2 DNA nucleotides). For single-material jobs, the material prefix can be omitted for all nucleotides (e.g., ACGUU for an RNA-only job or ACGTT for a DNA-only job), and nucleotides rU, mU, and dT are converted interchangeably as appropriate. For sequence design, sequence constraints can be specified using IUPAC degenerate nucleotide codes (see Table 1).

**Table 1:** IUPAC Degenerate Nucleotide Codes for RNA^a^

Code	Nucleotides
rM	rA or rC
rR	rA or rG
rW	rA or rU
rS	rC or rG
rY	rC or rU
rK	rG or rU
rV	rA, rC, or rG
rH	rA, rC, or rU
rD	rA, rG, or rU
rB	rC, rG, or rU
rN	rA, rC, rG, or rU

a For 2′OMe-RNA codes, “m” replaces “r” (e.g., “mS” instead of “rS”). For DNA codes, “d” replaces “r” and “T” replaces “U” (e.g., “dT” instead of “rU”). For mixed-material jobs, a nucleotide that can be either material has a wildcard prefix “w” (e.g., for an RNA/DNA job, “wS” is equivalent to “rS or dS”).

### Secondary Structure

A *secondary structure*, *s*, of one or more interacting nucleic acid strands is defined by a set of base pairs, each a Watson–Crick pair or a wobble pair (e.g., see Figure 1a).

• For RNA base pairs, Watson–Crick pairs are rA·rU and rC·rG and wobble pairs are rG·rU.
• For DNA base pairs, Watson–Crick pairs are dA·dT and dC·dG and there are no wobble pairs (dG and dT are considered to form mismatch dG × dT and not a wobble pair^11^).
• For 2′OMe-RNA base pairs, Watson–Crick pairs are mA·mU and mC·mG and wobble pairs are mG·mU.
• For RNA/DNA mixed-material base pairs, Watson–Crick pairs are rG·dC, dG·rC, rA·dT, dA·rU and wobble pairs are rG·dT; dG and rU are considered to form mismatch dG × rU and not a wobble pair.^12^
• For RNA/2′OMe-RNA mixed-material base pairs, Watson–Crick pairs are rG·mC, mG·rC, rA·mU, and mA·rU and wobble pairs are rG·mU and mG·rU.^13^

**Figure 1:**
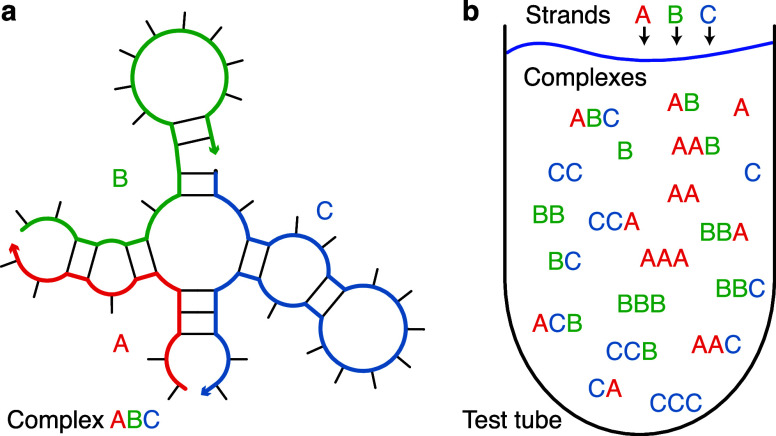
Complex and test tube ensembles. (a) A (connected unpseudoknotted) secondary structure for a complex of 3 strands with strand ordering π = ABC. An arrowhead denotes the 3′ end of each strand. (b) A test tube ensemble containing strand species Ψ^0^ = {A, B, C} interacting to form all complex species Ψ of up to *L*_max_ = 3 strands. Adapted from ref 9. Copyright 2020 American Chemical Society.

A *polymer graph* representation of a secondary structure is constructed by ordering the strands around a circle, drawing the backbones in succession from 5′ to 3′ around the circumference with a *nick* between each strand, and drawing straight lines connecting paired bases. A secondary structure is *unpseudoknotted* if there exists a strand ordering for which the polymer graph has no crossing lines, or *pseudoknotted* if all strand orderings contain crossing lines. A secondary structure is *connected* if no subset of the strands is free of (i.e., unpaired to) the others.^5^

Secondary structures may be specified in one of three ways for NUPACK calculations (see Table 2 for examples):

• *dot-parens-plus notation*: each unpaired base is represented by a dot, each base pair by matching parentheses, and each nick between strands by a plus.^1^
• *run-length encoded (RLE) dot-parens-plus notation*: as a shorthand for dot-parens-plus, any sequence of consecutive characters in dot-parens-plus may be replaced by the character followed by a number.^9^
• *DU+ notation*: Using DU+ notation, a duplex is denoted by D followed by the number of base pairs and an unpaired region is denoted by U followed by the number of unpaired nucleotides.^14^ Each duplex is followed immediately by the substructure (specified in DU+ notation) that is “enclosed” by the duplex. If this substructure includes more than one element, parentheses are used to denote scope. A nick between strands is specified by a “+”.

In mathematical expressions, it is convenient to represent secondary structure *s* using a *structure matrix S*(*s*) with entries *S* ^*a*,*b*^(*s*) = 1 if structure *s* contains base pair *a*·*b* and *S* ^*a*,*b*^(*s*) = 0 otherwise. Abusing notation, the entry *S* ^*a*,*a*^(*s*) = 1 if base *a* is unpaired in structure *s* and 0 otherwise. Hence, *S*(*s*) is a symmetric matrix with row and column sums of 1.

**Table 2:**
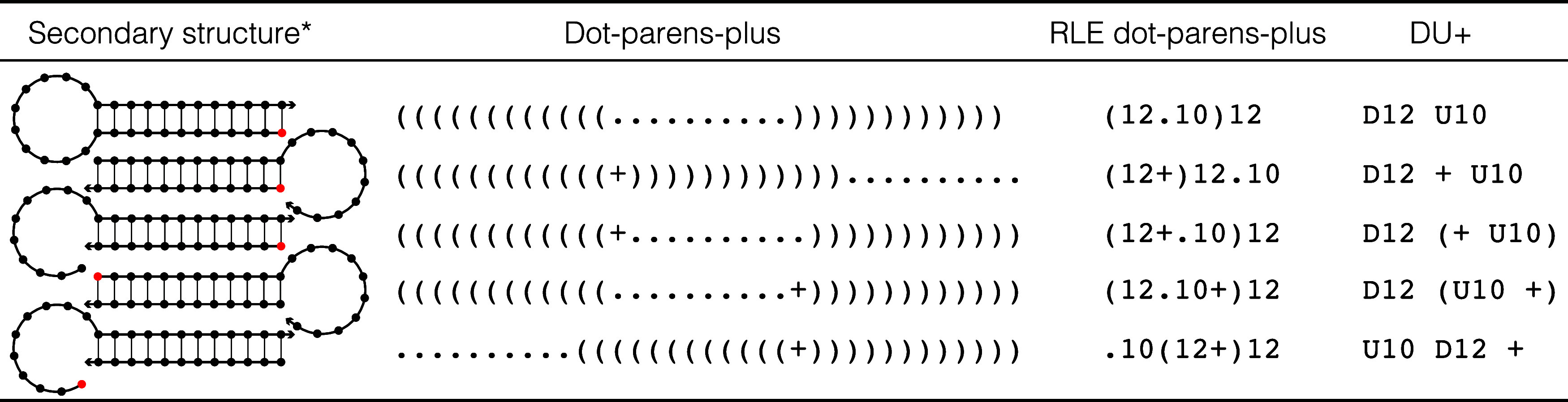
Secondary Structure Notation: Dot-Parens Plus, Run-Length Encoded (RLE) Dot-Parens-Plus, and DU+

* For each secondary structure, the first nucleotide is depicted in red.

### Complex Ensemble

Consider a complex of *L* distinct strands (each with a unique identifier in {1, ..., *L*}) corresponding to strand ordering π (e.g., see Figure 1a). The *complex ensemble* Γ­(ϕ) contains all connected polymer graphs with no crossing lines (i.e., all unpseudoknotted secondary structures).^5^ As a matter of algorithmic necessity, all of the dynamic programs in NUPACK operate on complex ensemble Γ­(ϕ) treating all strands as distinct. However, in the laboratory, strands with the same sequence are typically indistinguishable with respect to experimental observables. For comparison to experimental data, physical quantities calculated over ensemble Γ­(ϕ) are postprocessed to obtain the corresponding quantities calculated over *complex ensemble Γ*(ϕ) in which strands of the same species are treated as indistinguishable.^9^ The ensemble Γ­(ϕ) ⊆ Γ­(ϕ) is a maximal subset of distinct secondary structures for strand ordering π. Two secondary structures are indistinguishable if their polymer graphs can be rotated so that all strands are mapped onto indistinguishable strands, all base pairs are mapped onto base pairs, and all unpaired bases are mapped onto unpaired bases; otherwise the structures are distinct.^5^

### Test Tube Ensemble

A *test tube ensemble* is a dilute solution containing a set of strand species, Ψ^0^, introduced at user-specified concentrations, that interact to form a set of complex species, Ψ, each corresponding to a different strand ordering treating strands of the same species as indistinguishable (e.g., see Figure 1b).^5,9^ For *L* strands, there are (*L* – 1) strand orderings if all strands are different species (e.g., complexes π = ABC and π = ACB for *L* = 3 and strands A, B, C), but fewer than (*L* – 1) strand orderings if some strands are of the same species (e.g., complex π = AAA for *L* = 3 with three A strands). By the Representation Theorem,^5^ a secondary structure in the complex ensemble for one strand ordering does not appear in the complex ensemble for any other strand ordering, averting redundancy. It is often convenient to define Ψ to contain all complex species of up to *L*_max_ strands, although Ψ can be defined to contain arbitrary complex species formed from the strand species in Ψ^0^.

### Multitube Ensemble

Consider a multitube ensemble comprising the set of test tubes, Ω, where tube *h* ∈ Ω contains the set of strand species Ψ_*h*_^0^ interacting to form the set of complex species Ψ_*h*_. The set of all complexes in the multitube ensemble is then Ψ ≡ ∪_*h*∈Ω_Ψ_*h*_. Note that the multitube ensemble encompasses the complex and test tube ensembles as subsidiary special cases.^8^

### Loop-Based Free Energy Model

For each (unpseudoknotted connected) secondary structure *s* ∈ Γ­(ϕ), the free energy, $\overset{®}{\Delta G}\left(\phi ,s\right)$ , is estimated as the sum of the empirically determined free energies of the constituent loops^15–17^ plus a strand association penalty,^18^ Δ*G* ^assoc^, applied *L* – 1 times for a complex of *L* strands^5^

1 $$\overset{®}{\Delta G}\left(\phi ,s\right)=\left(L-1\right)\Delta G^{\mathrm{assoc}}+\sum_{\mathrm{loop}\in s}\Delta G\left(\mathrm{loop}\right)$$

The secondary structure of Figure 2 illustrates the different loop types, with loop free energy, Δ*G*(loop), modeled as follows:^9,17,19–22^

• A *hairpin loop* is closed by a single base-pair *i*·*j*. The loop free energy, Δ*G*_*i*,*j*_^hairpin^, depends on sequence and loop size.
• An *interior loop* is closed by two base pairs (*i*·*j* and *d*·*e* with *i* < *d* < *e* < *j*). The loop free energy, Δ*G*_*i*,*d*,*e*,*j*_^interior^ depends on sequence, loop size, and loop asymmetry. *Bulge loops* (where either *d* = *i* + 1 or *e* = *j* – 1) and *stack loops* (where both *d* = *i* + 1 and *e* = *j* – 1) are treated as special cases of interior loops.
• A *multiloop* is closed by three or more base pairs. The loop free energy is modeled as the sum of three material-dependent penalties: (1) Δ*G*_init_^multi^ for formation of a multiloop, (2) Δ*G*_bp_^multi^ for each closing base pair, (3) Δ*G*_nt_^multi^ for each unpaired nucleotide inside the multiloop; plus two sequence-dependent terms: (4) Δ*G*_*i*,*j*_^terminalbp^, a penalty for each closing pair *i*·*j*, (5) Δ*G*_stacking_, an optional coaxial and dangle stacking bonus.
• An *exterior loop* contains a nick between strands and any number of closing base pairs. The exterior loop free energy is the sum of Δ*G*_*i*,*j*_^terminalbp^ over all closing base pairs *i*·*j* plus an optional coaxial and dangle stacking bonus, Δ*G*_stacking_. Hence, an unpaired strand has a free energy of zero, corresponding to the reference state.^5^

See Sections S3–S5 of Reference 10 for details on the functional form of loop-based free energy models.

**Figure 2:**
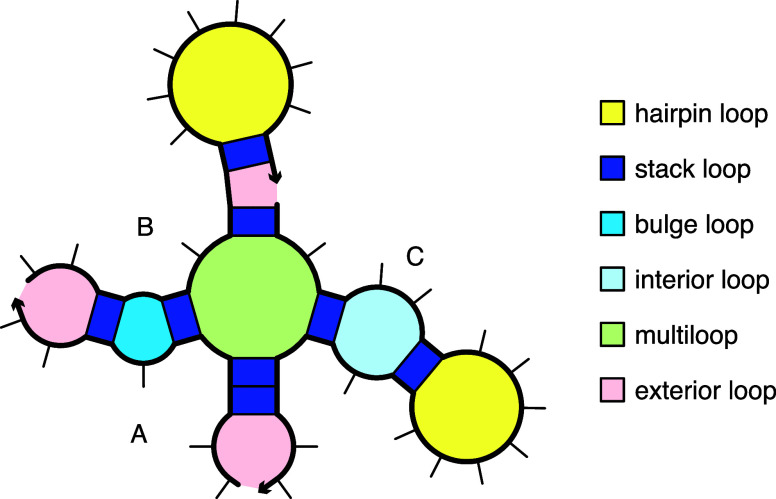
Loop-based free energy model for a complex. Canonical loop types for a complex with strand ordering π = ABC. Adapted from ref 9. Copyright 2020 American Chemical Society.

### Coaxial and Dangle Stacking

Within a multiloop or an exterior loop, there is a subensemble of coaxial stacking states between adjacent closing base pairs and dangle stacking states between closing base pairs and adjacent unpaired bases. Within a multiloop or exterior loop, a base pair can form one *coaxial stack* with an adjacent base pair, or can form a *dangle stack* with at most two adjacent unpaired bases; unpaired bases can either form no stack, or can form a dangle stack with at most one adjacent base pair (see Figure 3).

**Figure 3:**
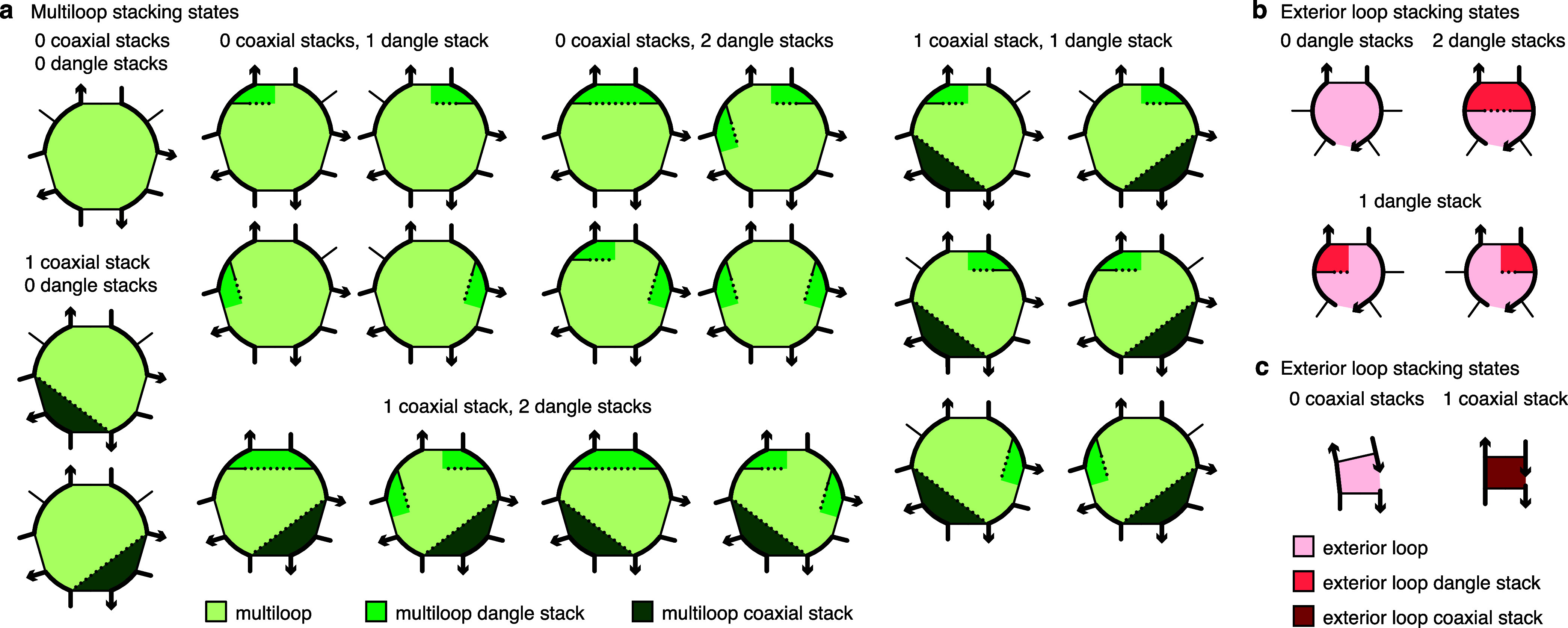
Coaxial and dangle stacking states for multiloops and exterior loops. (a) Stacking subensemble for the multiloop of Figure 2. (b, c) Stacking subensembes for two exterior loops from Figure 2. Adapted from ref 9. Copyright 2020 American Chemical Society.

For a given multiloop or exterior loop, the energetic contributions of all possible coaxial and dangle stacking states are enumerated so as to calculate the free energy^9^

2 $$\Delta G^{\mathrm{stacking}}=-\mathrm{kT}\;\log\sum_{\omega \in \mathrm{loop}}\prod_{x\in \omega }e^{-\Delta G_{x}/\mathrm{kT}}$$

where ω indexes the possible stacking states within the loop and *x* indexes the individual stacks (coaxial or dangle) within a stacking state. Here, *k* is the Boltzmann constant and *T* is temperature. The free energy of a multiloop or exterior loop is augmented by the corresponding Δ*G* ^stacking^ bonus. Hence, a secondary structure *s* continues to be defined as a set of base pairs, and the stacking states within a given multiloop or exterior loop are treated as a structural subensemble that contributes in a Boltzmann-weighted fashion to the free energy model for the loop. Let *s* ^∥^ ∈ *s* denote a stacking state of the paired and unpaired bases in *s*. We may equivalently define the free energy of secondary structure *s* in terms of the *stacking state free energies*

3 $$\overset{®}{\Delta G}\left(\phi ,s^{∥}\right)$$

for all stacking states *s* ^∥^ ∈ *s*

4 $$\overset{®}{\Delta G}\left(\phi ,s\right)=-\mathrm{kT}\;\log\sum_{s^{∥}\in s}e^{-\overset{®}{\Delta G}\left(\phi ,s^{∥}\right)/\mathrm{kT}}$$

Let Γ ^∥^(ϕ) denote the ensemble of stacking states corresponding to the complex ensemble of secondary structures Γ­(ϕ).

NUPACK supports the following coaxial and dangle stacking formulations:

• *All stacking* (default): complex ensemble with coaxial and dangle stacking.
• *Coaxial stacking:* complex ensemble with coaxial stacking.
• *Dangle stacking:* complex ensemble with dangle stacking.
• *No stacking:* complex ensemble without coaxial or dangle stacking.

### Symmetry Correction

For a secondary structure *s* ∈ Γ­(ϕ) with an *R*-fold rotational symmetry, there is an *R*-fold reduction in distinguishable conformational space, so the free energy $\overset{®}{\Delta G}\left(\phi ,s\right)$ must be adjusted^5^ by a symmetry correction

5 $$\Delta G\left(\phi ,s\right)=\overset{®}{\Delta G}\left(\phi ,s\right)+\Delta G^{\mathrm{sym}}\left(\phi ,s\right)$$

where

6 $$\Delta G^{\mathrm{sym}}\left(\phi ,s\right)=\mathrm{kT}\;\log R\left(\phi ,s\right)$$

Because the symmetry factor *R*(ϕ, *s*) is a global property of each secondary structure *s* ∈ Γ­(ϕ), it is not suitable for use with dynamic programs that treat multiple subproblems simultaneously without access to global structural information. As a result, dynamic programs operate on ensemble Γ­(ϕ) using physical model $\overset{®}{\Delta G}\left(\phi ,s\right)$ and then the Distinguishability Correction Theorem^5^ enables exact conversion of physical quantities to ensemble Γ­(ϕ) using physical model Δ*G*(ϕ, *s*). Interestingly, ensembles Γ­(ϕ) and Γ­(ϕ) both have utility when examining the physical properties of a complex as they provide related but different perspectives, akin to complementary thought experiments.^9^

### Free Energy Parameters

NUPACK supports temperature-dependent parameter sets with nearest-neighbor sequence dependence that provide loop free energies and enthalpies for single-material jobs (RNA, DNA, or 2′OMe-RNA), including:

• rna06: RNA parameters based on (Mathews et al.),^20^ (Mathews et al.),^23^ and (Lu et al.)^21^ with additional parameters^19,24^ including coaxial stacking^20,22^ and dangle stacking^16,22,24^ in a user-specified concentration of Na^+^.
• dna04.2: DNA parameters based on (SantaLucia)^17^ and (SantaLucia and Hicks)^11^ with additional parameters^24^ including coaxial stacking,^25^ dangle stacking,^24,26^ GT internal mismatch values,^11,27–31^ internal asymmetry values,^11^ and terminal mismatch values^22,32^ in user-specified concentrations of Na^+^, K^+^, NH_4_^+^, and Mg^2+^.^11,17,25,33^

• merna06: 2′OMe-RNA parameters for calculations in 0.12 M Na^+^ based on (Kierzek et al.)^13^ using stack loop, coaxial stacking, terminal base pair, and strand association values from rna-merna06 and all other values from rna06.

or mixed-material jobs (RNA/DNA or RNA/2′OMe-RNA), including:

• rna-dna06: RNA/DNA parameters for hybrid stack loops,^34^ hybrid internal mismatches,^12,35^ and chimeric stack loops^36^ with additional parameters for mixed-material loops;^10^ for use in conjunction with single-material parameter sets rna06 and dna04.2 in a user-specified concentration of Na^+^.
• rna-merna06: RNA/2′OMe-RNA parameters based on hybrid stack loops^13^ with additional parameters for mixed-material loops;^10^ for use in conjunction with single-material parameter sets rna06 and merna06 in 0.12 M Na^+^.

Free energies are expressed in kcal/mol.

### Historical Options

For backward compatibility with NUPACK 3, NUPACK 4 also supports historical stacking formulations (without coaxial stacking and with approximate dangle stacking) and parameter sets. The default physical models for NUPACK 3 can be reproduced as follows:

• For RNA: *Parameters*: rna95 (NUPACK3); *Ensemble*: Some dangles (NUPACK3).
• For DNA: *Parameters*: dna04 (NUPACK3); *Ensemble*: Some dangles (NUPACK3).

### Salts

NUPACK supports the following salt conditions:

• For RNA single-material jobs using rna06: *Sodium*: the concentration of sodium ions, [Na^+^], is specified in units of molar (default: 1.0, range: [0.05,1.0]);^10^ *Magnesium*: only 0.0 M [Mg^2+^] is supported.
• For DNA single-material jobs using dna04.2: *Sodium*: the sum of the concentrations of (monovalent) sodium, potassium, and ammonium ions, [Na^+^] + [K^+^] + [NH_4_^+^], is specified in units of molar (default: 1.0, range: [0.05,1.1]);^11,17^ *Magnesium*: the concentration of (divalent) magnesium ions, [Mg^2+^], is specified in units of molar (default: 0.0, range: [0.0,0.2]).^25,33^

• For 2′OMe-RNA single-material jobs using merna06, the only supported salt conditions^13^ are 0.12 M [Na^+^].
For RNA/DNA mixed-material jobs using rna-dna06: *Sodium*: the concentration of sodium ions, [Na^+^], is specified in units of molar (default: 1.0, range: [0.12,1.0]);^10^ *Magnesium*: only 0.0 M [Mg^2+^] is supported.
• For RNA/2′OMe-RNA mixed-material jobs using rna-merna06, the only supported salt conditions^13^ are 0.12 M [Na^+^].

## Physical Quantities

Consider a multitube ensemble comprising the set of test tubes, Ω, where tube *h* ∈ Ω contains a set of strand species Ψ_*h*_^0^ interacting to form a set of complex species Ψ_*h*_. Let *j* ∈ Ψ_*h*_ denote a complex with sequence ϕ_*j*_ and complex ensembles Γ­(ϕ_*j*_) (treating all strands as distinct) and Γ­(ϕ_*j*_) (treating strands of the same species as indistinguishable). NUPACK calculates a number of physical quantities over these ensembles.^5,9^

### Partition Function

For complex *j*, the *partition function* evaluated over ensemble Γ­(ϕ_*j*_) treating strands of the same species as indistinguishable is

7 $$Q\left(\phi_{j}\right)=\sum_{s\in \Gamma \left(\phi_{j}\right)}e^{-\Delta G\left(\phi_{j},s\right)/kT}$$

### Complex Free Energy

For complex *j*, the *complex free energy* is

8 $$\Delta G\left(\phi_{j}\right)\equiv -\mathrm{kT}\;\log\left(Q\left(\phi_{j}\right)\right)$$

### Secondary Structure Free Energy

For complex *j*, the *secondary structure free energy* for *s* treating strands of the same species as indistinguishable is Δ*G*(ϕ_*j*_, *s*), given by ([Disp-formula eq5]).

### Equilibrium Structure Probability

For complex *j*, the *equilibrium structure probability* of any secondary structure *s* ∈ Γ­(ϕ_*j*_) treating strands of the same species as indistinguishable is

9 $$p\left(\phi_{j},s\right)=e^{-\Delta G\left(\phi_{j},s\right)/kT}/Q\left(\phi_{j}\right)$$

### Boltzmann-Sampled Structures

For complex *j*, a set of *J Boltzmann-sampled structures* from ensemble Γ­(ϕ_*j*_) treating strands of the same species as indistinguishable is denoted

10 $$\Gamma_{\mathrm{sample}}\left(\phi_{j},J\right)\in \Gamma \left(\phi_{j}\right)$$

Boltzmann-sampled structures are available only via the NUPACK Python module.

### Equilibrium Base-pairing Probabilities

For complex *j*, the base-pairing probability matrix *P̅*(ϕ_*j*_) has entries *P̅* ^*a*,*b*^(ϕ_*j*_) ∈ [0, 1] corresponding to the *equilibrium base-pairing probability*

11 $${\mathrm{P̅}}^{a,b}\left(\phi_{j}\right)=\sum_{s\in \mathrm{\Gamma ̅}\left(\phi_{j}\right)}\mathrm{p̅}\left(\phi_{j},s\right)S^{a,b}\left(s\right)$$

representing the equilibrium probability that base pair *a*·*b* forms within ensemble Γ̅(ϕ_*j*_), treating all strands as distinct. Here, *S*(*s*) is the structure matrix and *p̅*(*s*) is the equilibrium probability of structure *s* ∈ Γ̅(ϕ_*j*_) treating all strands as distinct. Abusing notation, the entry *P̅* ^*a,a*^(ϕ_*j*_) ∈ [0, 1] denotes the equilibrium probability that base *a* is unpaired over ensemble Γ­(ϕ_*j*_). Hence, *P̅*(ϕ_*j*_) is a symmetric matrix with row and column sums of 1. In NUPACK graphics, the diagonal entries denoting unpaired bases are depicted as an extra column to the right of the matrix.

### MFE Proxy Structure

For complex *j*, the minimum free energy (MFE) stacking state *s*_MFE_^∥^(ϕ_*j*_) ∈ Γ ^∥^(ϕ_*j*_) treating all strands as distinct is

12 $$s_{\mathrm{MFE}}^{∥}\left(\phi_{j}\right)=\arg\min_{s^{∥}\in {\mathrm{\Gamma ̅}}^{∥}\left(\phi_{j}\right)}\overset{®}{\Delta G}\left(\phi_{j},s^{∥}\right)$$

The corresponding *MFE proxy structure* is

$$s_{\mathrm{MFE}\prime }\left(\phi_{j}\right)\equiv \left\{s\in \mathrm{\Gamma ̅}(\phi_{j})\;|\;s_{\mathrm{MFE}}^{∥}\left(\phi_{j}\right)\in s\right\}$$ 13

defined as the secondary structure containing the MFE stacking state within its subensemble. The free energy of the MFE proxy structure is reported as

14 $$\Delta G\left(\phi_{j},s_{\mathrm{MFE}\prime }\left(\phi_{j}\right)\right)$$

treating strands of the same species as indistinguishable, which satisfies the inequality

15 $$\Delta G\left(\phi_{j},s_{\mathrm{MFE}\prime }\left(\phi_{j}\right)\right)\geq \Delta G\left(\phi_{j}\right)$$

relative to the complex free energy. There may be more than one MFE stacking state, each corresponding to the same or different MFE proxy structures. If there are multiple MFE proxy structures, the NUPACK web app presents only one of them; to see multiple MFE proxy structures, use the NUPACK Python module. The NUPACK Python module also provides the option to perform the structure search of eq 13 over ensemble Γ­(ϕ_*j*_) instead of Γ̅(ϕ_*j*_), so that the MFE proxy structure is determined treating strands of the same species as indistinguishable.

### Suboptimal Proxy Structures

For complex *j*, the set of *suboptimal proxy structures* with stacking states within a specified Δ*G*_gap_ ≥ 0 of the MFE stacking state is denoted as

16 $${\mathrm{\Gamma ̅}}_{\mathrm{subopt}}\left(\phi_{j},\Delta G_{\mathrm{gap}}\right)=\{s\in \mathrm{\Gamma ̅}\left(\phi_{j}\right)\;|\;s^{∥}\in s,\overset{®}{\Delta G}\left(\phi_{j},s^{∥}\right)\leq \overset{®}{\Delta G}\left(\phi_{j},s_{\mathrm{MFE}}^{∥}\left(\phi_{j}\right)\right)+\Delta G_{\mathrm{gap}}\}$$

Suboptimal proxy structures are available only via the NUPACK Python module, including the option to perform the structure search of eq 16 over ensemble Γ­(ϕ_*j*_) instead of Γ̅(ϕ_*j*_), so that the MFE proxy structure and the suboptimal proxy structures in the requested energy gap are determined treating strands of the same species as indistinguishable.

### Complex Ensemble Defect

For complex *j* with target structure *s*_*j*_, the *complex ensemble defect*

17 $$n\left(\phi_{j},s_{j}\right)=\left|\phi_{j}\right|-\sum_{\begin{array}{l} 1\leq a\leq \left|\phi_{j}\right|,\\ 1\leq b\leq \left|\phi_{j}\right| \end{array}}{\mathrm{P̅}}^{a,b}\left(\phi_{j}\right)S^{a,b}\left(s_{j}\right)$$

represents the equilibrium number of incorrectly paired nucleotides over the ensemble Γ̅(ϕ_*j*_) relative to target structure *s*_*j*_.^3,6^ Here, *P̅*(ϕ_*j*_) is the equilibrium base-pairing probability matrix and *S*(*s*_*j*_) is the target structure matrix for *s*_*j*_. The *normalized complex ensemble defect* is then

18 $$N_{j}\equiv n\left(\phi_{j},s_{j}\right)/\left|\phi_{j}\right|\in \left[0,1\right]$$

representing the equilibrium fraction of incorrectly paired nucleotides evaluated over the ensemble of complex *j* relative to target structure *s*_*j*_.

### Complex Ensemble Size

For complex *j*, the *number of secondary structures* in the complex ensemble, treating all strands as distinct, is

19 $$\left|\mathrm{\Gamma ̅}\left(\phi_{j}\right)\right|$$

The corresponding *number of stacking states* is

20 $$\left|{\mathrm{\Gamma ̅}}^{∥}\left(\phi_{j}\right)\right|$$

### Equilibrium Complex Concentrations

For the set of complexes Ψ_*h*_ in test tube *h*, the set of *equilibrium complex concentrations* is denoted

21 $$x_{\Psi_{h}}\equiv x_{j},\forall j\in \Psi_{h}$$

These concentrations are the unique solution to the strictly convex optimization problem^5^

22 $$\min_{x_{\Psi_{h}}}\sum_{j\in \Psi_{h}}x_{j}\left(\log x_{j}-\log Q_{j}-1\right)$$

23 $$\mathrm{subject}\;\mathrm{to}\sum_{j\in \Psi_{h}}A_{i,j}x_{j}=x_{i}^{0},\forall i\in \Psi_{h}^{0}$$

expressed in terms of the previously calculated set of partition functions *Q*_*Ψh*_. Here, the constraints impose conservation of mass: *A* is the stoichiometry matrix such that *A*_*i*,*j*_ is the number of strands of type *i* in complex *j*, and *x*_*i*_^0^ is the total concentration of strand *i* present in the test tube. Based on dimensional analysis,^5,10^ the convex optimization problem is formulated in terms of mole fractions, but for convenience, NUPACK accepts molar strand concentrations, [*i*]^0^ = *x*_*i*_^0^ρ_H_2_O_, as inputs and returns molar complex concentrations, [*j*] = *x*_*j*_ρ_H_2_O_, as outputs, where ρ_H_2_O_ is the molarity of water. Hence, the user specifies the set of molar strand concentrations [*i*]^0^ ∀*i* ∈ Ψ_*h*_^0^ and NUPACK calculates the set of molar complex concentrations [*j*] ∀*j* ∈ Ψ_*h*_.

### Test Tube Fraction of Bases Unpaired

For a test tube *h* ∈ Ω containing the set of complexes Ψ_*h*_, the *test tube fraction of bases unpaired*

24 $$f_{h}^{\mathrm{unpaired}}\in \left[0,1\right]$$

denotes the fraction of bases that are unpaired in tube *h* at equilibrium, which is calculated based on the set of equilibrium concentrations *x*_*Ψh*_ and the set of base-pairing probability matrices *P* _Ψ_*h*__.

### Test Tube Ensemble Pair Fractions

For a test tube *h* ∈ Ω containing the set of complexes Ψ_*h*_, the *test tube ensemble pair fraction*

25 $$f_{h}^{A}\left(a_{A}\cdot b_{B}\right)$$

denotes the fraction of A strands that form base pair *a*_A_·*b*_B_ in tube *h*. Correspondingly,

26 $$f_{h}^{B}\left(a_{A}\cdot b_{B}\right)$$

denotes the fraction of B strands that form base pair *a*_A_·*b*_B_ in tube *h*. These base-pairing observables depend on the set of equilibrium concentrations *x*_*Ψh*_ and the set of base-pairing probability matrices *P* _Ψ_*h*__. The number of distinct bases in the test tube is

27 $$N_{\mathrm{distinct}}\equiv \sum_{i=1}^{\left|\Psi_{h}^{0}\right|}\left|\phi_{i}\right|$$

representing the total number of bases in all |Ψ_*h*_^0^| strand species. Numbering the distinct bases from 1 to *N*_distinct_, the ensemble pair fractions are then stored as an (asymmetric) *N*_distinct_ × *N*_distinct_ matrix with the fraction of each nucleotide that is unpaired stored on the diagonal. Hence, the matrix of test tube ensemble pair fractions is asymmetric with row and column sums of 1. In NUPACK graphics, the diagonal entries denoting unpaired bases are depicted as an extra column to the right of the matrix.

### Test Tube Ensemble Defect

Consider test tube *h* ∈ Ω containing a set of desired *on-target complexes*, Ψ_*h*_^on^, and a set of undesired *off-target complexes*, Ψ_*h*_^off^. The set of complexes in the test tube is then:

28 $$\Psi_{h}=\Psi_{h}^{\mathrm{on}}\cup \Psi_{h}^{\mathrm{off}}$$

Let each on-target complex, *j* ∈ Ψ_*h*_^on^, have a user-specified target secondary structure, *s*_*j*_, and a user-specified target concentration, *y*_*h*,*j*_. Let each off-target complex, *j* ∈ Ψ_*h*_^off^, have a vanishing target concentration (*y*_*h*,*j*_ = 0) and no target structure (*s*_*j*_ = Ø). The *test tube ensemble defect*,

29 $$C\left(\phi_{\Psi_{h}},s_{\Psi_{h}},y_{h,\Psi_{h}}\right)=\sum_{j\in \Psi_{h}^{\mathrm{on}}}\left[n\left(\phi_{j},s_{j}\right)\;\min\left(x_{h,j},y_{h,j}\right)+\left|\phi_{j}\right|\;\max\left(y_{h,j}-x_{h,j},0\right)\right]$$

represents the equilibrium concentration of incorrectly paired nucleotides over the ensemble of test tube *h*.^7^ Here, *x*_*h*,*j*_ is the equilibrium concentration of complex *j* in tube *h*. For each on-target complex, *j* ∈ Ψ_*h*_^on^, the first term in the sum represents the *structural defect*, quantifying the concentration of nucleotides that are in an incorrect base-pairing state within the ensemble of complex *j*, and the second term in the sum represents the *concentration defect*, quantifying the concentration of nucleotides that are in an incorrect base-pairing state because there is a deficiency in the concentration of complex *j*. For each off-target complex, *j* ∈ Ψ_*h*_^off^, the structural and concentration defects are identically zero, since *y*_*h*,*j*_ = 0. This does not mean that the defects associated with off-targets are ignored. By conservation of mass, nonzero off-target concentrations imply deficiencies in on-target concentrations, and these concentration defects are quantified by the equation above.^7^ The *normalized test tube ensemble defect* is then denoted

30 $$M_{h}\equiv C_{h}/y_{h}^{\mathrm{nt}}\in \left[0,1\right]$$

representing the equilibrium fraction of incorrectly paired nucleotides in tube *h*. Here,

31 $$y_{h}^{\mathrm{nt}}\equiv \sum_{j\in \Psi_{h}^{\mathrm{on}}}\left|\phi_{j}\right|y_{h,j}$$

is the total concentration of nucleotides in tube *h*. As $M_{h}$ approaches zero, each on-target complex, *j* ∈ Ψ_*h*_^on^, approaches its target concentration, *y*_*h*,*j*_, and is dominated by its target structure, *s*_*j*_, and each off-target complex, *j* ∈ Ψ_*h*_^off^, forms with vanishing target concentration.

### Multitube Ensemble Defect

For the set of test tubes Ω, the *multitube ensemble defect*,

32 $$M\equiv \frac{1}{\left|\Omega \right|}\sum_{h\in \Omega }M_{h}\in \left(0,1\right)$$

represents the average equilibrium fraction of incorrectly paired nucleotides over the set of test tubes Ω.

## Design Formulation

NUPACK provides a framework for engineering reaction pathways for dynamic hybridization cascades (e.g., shape and sequence transduction using small conditional RNAs^37,38^ ) or for engineering pseudoknotted structures (e.g., RNA origamis^39^). In either case, sequence design is performed over a multitube ensemble (see Figure 4 for a cautionary tale emphasizing the advantages of test tube design over complex design).^7,8^

**Figure 4:**
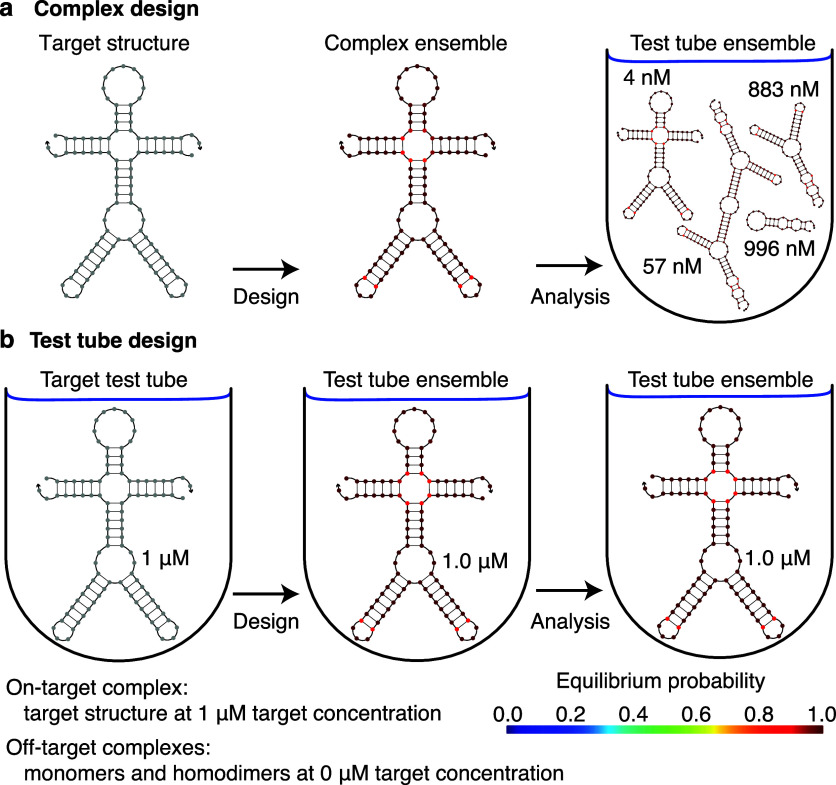
A cautionary tale: the advantages of test tube design over complex design. (a) Complex design. Sequence design formulated in the context of a complex (left) ensures that at equilibrium the target structure dominates the structural ensemble of the complex (center). Unfortunately, subsequent test tube analysis reveals that the desired on-target complex occurs at negligible concentration relative to other undesired off-target complexes (right). With complex design, neither the concentration of the desired on-target complex, nor the concentrations of undesired off-target complexes are considered. As a result, sequences that are successfully optimized to predominantly adopt a target secondary structure in the context of an on-target complex, may nonetheless fail to ensure that this complex forms at appreciable concentration when the strands are introduced into a test tube. (b) Test tube design. Sequence design formulated in the context of a test tube (left) ensures that at equilibrium the desired on-target complex is dominated by its target structure and forms at approximately its target concentration, and that undesired off-target complexes form at negligible concentrations (center). Subsequent test tube analysis (right) provides no new information and no unpleasant surprises since the design and analysis ensembles are identical. Adapted from ref 7. Copyright 2014 American Chemical Society.

For reaction pathway engineering, sequence design is formulated as a multistate optimization problem using a set of target test tubes to represent elementary steps of the reaction pathway, as well as to model crosstalk between components. Note that *kinetic design* of a test tube ensemble is achieved by performing *equilibrium optimization* of a multitube ensemble: each target test tube isolates different subsets of components in local equilibrium, enabling optimization of kinetically significant states that would appear insignificant if all components were allowed to interact in a single ensemble.

For engineering structures including the possibility of pseudoknots, each target test tube contains only complex ensembles comprising unpseudoknotted structures, but by imposing sequence constraints between tubes, it is possible to collectively impose pseudoknotted design requirements.

In a multitube design ensemble, each target test tube contains a set of desired *on-target complexes*, each with a target secondary structure and target concentration, and a set of undesired *off-target complexes*, each with vanishing target concentration. Optimization of the *multitube ensemble defect*, representing the average equilibrium fraction of incorrectly paired nucleotides over the design ensemble, implements both a positive design paradigm (designing for desired properties) and a negative design paradigm (designing against unwanted properties). *Defect weights* can be specified to prioritize or deprioritize design quality for different portions of the design ensemble. Sequence design is performed subject to user-specified *hard constraints* that prohibit sequences violating the constraints and *soft constraints* that penalize (but do not prohibit) suboptimal sequences.

### Reaction Pathways

Consider a set of nucleic acid molecules intended to execute a prescribed hybridization cascade.^8^ For example, the reaction pathway of Figure 5 describes small conditional RNAs (scRNAs) that upon binding to input X, perform shape and sequence transduction to form a Dicer substrate targeting an independent output Y for silencing.^37^ A *reaction pathway* specifies the *elementary steps* (each a self-assembly or disassembly operation in which complexes form or break) by which the molecules are intended to interact, the desired secondary structure for each on-pathway complex, and the complementarity relationships between sequence domains in the molecules. In the reaction pathway of Figure 5, there are two elementary steps (Step 1: X + A·B → X·A + B, Step 2: B + C → B·C) involving six on-pathway complexes (X, A·B, X·A, B, C, B·C) and numerous sequence domains (“a*” reverse complementary to “a”, “b*” reverse complementary to “b”, and so on).

**Figure 5:**
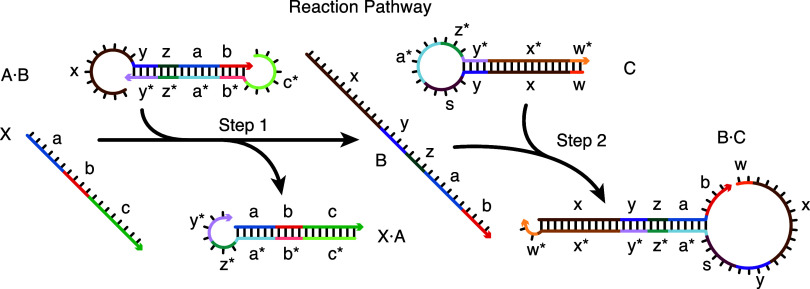
Reaction pathway schematic. Conditional Dicer substrate formation via shape and sequence transduction with small conditional RNAs (scRNAs).^37^ scRNA A·B detects input X (comprising sequence “a-b-c”), leading to production of Dicer substrate B·C (targeting independent sequence “w-x-y-z”). Step 1: X displaces A from B via toehold-mediated 3-way branch migration and spontaneous dissociation. Step 2: B assembles with C via loop/toehold nucleation and 3-way branch migration to form Dicer substrate B·C. See ref 8 for additional reaction pathway case studies. Adapted from ref 8. Copyright 2017 American Chemical Society.

In addition to specifying a set of desired on-pathway elementary steps, each reaction pathway also implicitly specifies a much larger set of off-pathway interactions, corresponding to undesired *crosstalk* between components within the pathway or with components from other unrelated reaction pathways. To perform sequence design for reaction pathway engineering, we formulate a multistate optimization problem to explicitly design for on-pathway elementary steps (a positive design paradigm) and against off-pathway crosstalk (a negative design paradigm).^8^

### Multitube Design Ensemble

A multitube design problem is specified as a set of *target test tubes*, Ω.^8^ Each tube, *h* ∈ Ω, contains a set of desired *on-target complexes*, Ψ_*h*_^on^, and a set of undesired *off-target complexes*, Ψ_*h*_^off^. For each on-target complex, *j* ∈ Ψ_*h*_^on^, the user specifies a target secondary structure, *s*_*j*_, and a target concentration, *y*_*h*,*j*_. For each off-target complex, *j* ∈ Ψ_*h*_^off^, the target concentration is vanishing (*y*_*h*,*j*_ = 0) and there is no target structure (*s*_*j*_ = Ø). Note that complex *j* may have a different target concentration, *y*_*h*,*j*_, in each tube *h* (e.g., may be an on-target in one tube and an off-target in another tube). By contrast, complex *j* has the same target structure, *s*_*j*_, in all tubes where it appears as an on-target (the target structure is ignored in any tubes where complex *j* appears as on off-target).

The set of complexes in tube *h* is then Ψ_*h*_ ≡ Ψ_*h*_^on^ ∪ Ψ_*h*_^off^ and the set of all complexes in multistate test tube ensemble Ω is Ψ ≡ ∪_*h*∈Ω_Ψ_*h*_. Let

33 $$\phi_{\Psi }\equiv \phi_{j},\forall j\in \Psi$$

denote the set of sequences for the complexes in Ψ.

Consider specification of the multitube ensemble, Ω, for the design of *N* orthogonal systems for a reaction pathway of *M* elementary steps. One *elementary step tube* is specified for each step *m* = 0, ..., *M* for each system *n* = 1, ..., *N* (treating formation of the initial reactants as a precursor “Step 0”). Additionally, a single *global crosstalk tube* is specified to minimize off-pathway interactions between the reactive species generated during all elementary steps of all systems. The total number of target test tubes is then |Ω| = *N* × (*M* + 1) + 1.

### Target Test Tubes

Figure 6 depicts target test tubes for the reaction pathway of Figure 5. There are three *elementary step tubes*, each containing on-target complexes corresponding to the products of the corresponding step: the Step 0 tube contains on-targets X, A·B, and C; the Step 1 tube contains on-targets X·A and B; the Step 2 tube contains on-target B·C. Each elementary step tube contains a set of on-target complexes (each with a target secondary structure and target concentration), corresponding to the on-pathway hybridization products for a given step, and a set of undesired off-target complexes (each with vanishing target concentration), corresponding to on-pathway reactants and off-pathway hybridization crosstalk for a given step. Hence, these elementary step tubes design for full conversion of cognate reactants into cognate products and against local crosstalk between these same reactants.

**Figure 6:**
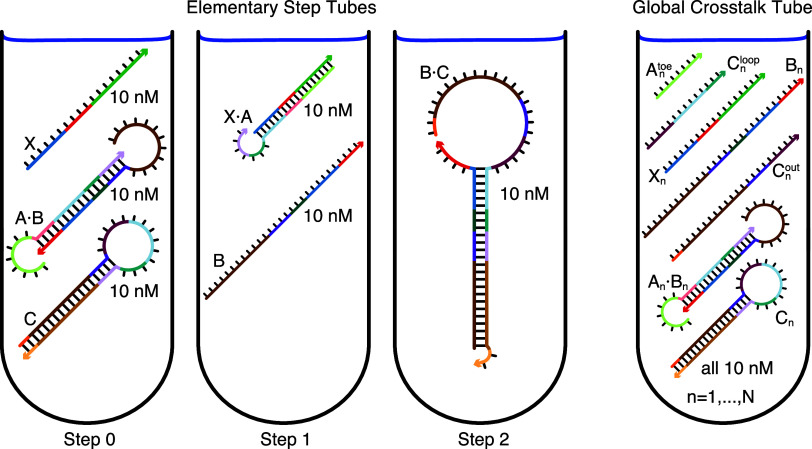
Target test tubes. Left: Elementary step tubes. Step 0 tube: target X and scRNAs A·B and C. Step 1 tube: X·A and B. Step 2 tube: Dicer substrate B·C. Each target test tube contains the depicted on-target complexes corresponding to the on-pathway products for a given step (each with the depicted target secondary structure and a target concentration of 10 nM) as well as off-target complexes (not depicted) corresponding to on-pathway reactants and off-pathway crosstalk for a given step. To design *N* orthogonal systems, there are three elementary step tubes for each system *n* = 1, ..., *N*. Right: Global crosstalk tube. Contains the depicted on-target complexes corresponding to reactive species generated during Steps 0, 1, 2 as well as off-target complexes (not depicted) corresponding to off-pathway interactions between these reactive species. To design *N* orthogonal systems, the global crosstalk tube contains a set of on-targets and off-targets for each system *n* = 1, ..., *N*. Adapted from ref 8. Copyright 2017 American Chemical Society.

To simultaneously design *N* orthogonal systems, three elementary step tubes of the type shown in Figure 6 (left) are specified for each system. Furthermore, to design against off-pathway interactions within and between systems, a single *global crosstalk tube* is specified (right). In the global crosstalk tube, the on-target complexes correspond to all reactive species generated during all elementary steps (*m* = 0, 1, 2) for all systems (*n* = 1, ..., *N*); the off-target complexes correspond to noncognate interactions between these reactive species. Crucially, the global crosstalk tube ensemble omits the cognate products that the reactive species are intended to form (they appear as neither on-targets nor off-targets). Hence, all reactive species in the global crosstalk tube are forced to either perform no reaction (remaining as desired on-targets) or undergo a crosstalk reaction (forming undesired off-targets), providing the basis for minimization of global crosstalk during sequence optimization. To design 8 orthogonal systems for this reaction pathway, the total number of target test tubes is then |Ω| = 8 × 3 + 1 = 25. See section S2.2 of ref 8 for a general description of how to specify target test tubes for a given reaction pathway and a number of illustrative case studies.

Note that each target test tube isolates a different subset of the system components in local equilibrium, enabling optimization of kinetically significant states that would appear insignificant if all components were allowed to interact in a single ensemble. For example, the Step 1 tube in Figure 6 simultaneously optimizes for high-yield production of unstructured intermediate B and against appreciable formation of off-target dimer B·B, promoting rapid nucleation of the unstructured toehold in B with the loop of hairpin C during the next step of the reaction pathway.

Note also that for a tube containing a given set of system components, the cognate products of their interactions can be excluded from the ensemble (appearing as neither on-targets nor off-targets), enabling optimization for high-yield well-structured reactants and against crosstalk. For example, in Figure 6, the Step 0 tube excludes the cognate product of Step 1 (X·A) from the ensemble in order to optimize formation of initial reactants X, A·B, and C and discourage competing crosstalk interactions (e.g., X·X, A·A, X·C).

### Design Objective Function

The design objective function is the multitube ensemble defect,^8^ M∈[0,1] of eq 32, representing the average equilibrium fraction of incorrectly paired nucleotides over the multitube ensemble, Ω. Test tube ensemble defect optimization implements a positive design paradigm (stabilize on-targets) and a negative design paradigm (destabilize off-targets) at two levels: a) designing for the on-target structure and against all off-target structures within the structural ensemble of each on-target complex,^3,6^ and b) designing for the target concentration of each on-target complex and against the formation of all off-target complexes within the ensemble of the test tube.^7,8^ Both paradigms are crucial at both levels in order to achieve high-quality test tube designs with a low test tube ensemble defect.^7,8^

### Defect Weights

To prioritize or deprioritize design quality for a portion of the design ensemble, the defect-weighted objective function, $M_{W}$ , incorporates user-specified defect weights, W , for the multitube ensemble or for any domain, strand, complex, or tube. With the default value of unity for all weights, $M_{W}$ is simply the multitube ensemble defect, M . With custom defect weights in the range [0, ∞), the physical meaning of the objective function is distorted in the service of adjusting design priorities. Increasing the weight for a tube, complex, strand or domain will lead to a corresponding increase in the allocation of effort to designing this entity, typically leading to a corresponding reduction in the defect contribution of the entity. Likewise, decreasing the weight for a domain, strand, complex, or tube will lead to a corresponding decrease in the allocation of effort to designing this entity, typically leading to a corresponding increase in the defect contribution of the entity.

### Hard Constraints

Sequence design is performed subject to user-specified *hard constraints* that prohibit sequences violating the constraints. NUPACK supports the following types of hard constraints:^8,9^

• *Assignment constraints:* Constrain consecutive nucleotides to have a specified sequence (specified 5′ to 3′ using degenerate nucleotide codes; see Table 1); specify an assignment constraint by defining a *domain*.
• *Match constraints:* Force a concatenation of one list of domains and an equal-length concatenation of another list of domains to have identical sequences.
• *Complementarity constraints:* Force a concatenation of one list of domains to be the reverse Watson–Crick complement of an equal-length concatenation of another list of domains (optionally allow wobble complements). Note that nucleotides that are base-paired in the target structure of an on-target complex are automatically assigned a complementarity constraint.
• *Diversity constraints:* Force every word of a specified length to contain a specified degree of sequence diversity, either globally or for a concatenated list of domains (e.g., require every subsequence of length 4 to have at least 2 nucleotide types).
• *Similarity constraints:* Force a concatenation of a list of domains to match an equal-length reference sequence to within a specified fractional range (e.g., require 45%–55% GC content by imposing a similarity constraint of 45%–55% to a sequence that is poly-S).
• *Window constraints:* Force a concatenation of a list of domains to be a subsequence of a source sequence (e.g., the source sequence is an mRNA), or more generally, a subsequence of one of multiple source sequences.
• *Library constraints:* Force a concatenation of a list of domains to have sequences drawn from a concatenated list of libraries. Each library contains a set of alternative sequences of equal length (e.g., a library of toehold sequences or a library of codons).
• *Pattern constraints:* Prevent a list of patterns from appearing globally or in a concatenation of a list of domains (e.g., prevent GGGG, which is prone to forming G-quadruplexes that are not accounted for in the empirical physical model).

Let R denote the user-specified set of hard constraints for a design problem.

### Soft Constraints

Additionally, the user may specify *soft constraints* that use auxiliary objective functions,

$$w_{k}\;f_{k}\left(\phi_{\Psi }\right)$$ 34

to penalize (rather than prohibit) suboptimal sequences during the design process.^40^ Here, *f*_*k*_(ϕ_Ψ_) ∈ [0, 1] is the penalty function for soft constraint *k* and *w*_*k*_ ∈ [0, ∞) (default: 1) is the corresponding user-specified weight. Soft constraints can reduce design cost relative to the corresponding hard constraint by making it easier for the optimization process to identify candidate sequence mutations. Soft constraints can also increase flexibility by enabling specification of new design goals (e.g., designing two toeholds to have comparable binding strength) for which there is no hard constraint analog. NUPACK supports the following types of soft constraints:^40^

• *Similarity constraints:* Penalize a concatenation of a list of domains if it does not match an equal-length reference sequence to within a specified fractional range (e.g., prioritize 45%–55% GC content by imposing a similarity constraint of 45%–55% to a sequence that is poly-S).
• *Pattern constraints:* Penalize a list of patterns if they appear globally or in a concatenation of a list of domains (e.g., penalize GGGG, which is prone to forming G-quadruplexes that are not accounted for in the empirical physical model).
• *Symmetry constraints:* Penalize a subsequence of a specified word length:^41^ (1) if the word appears in more than one location in the design, (2) if the reverse complement of the word appears elsewhere in a location that is not intended to form a duplex with the word, or (3) if the word is self-complementary.
• *Energy match constraints:* Penalize a set of duplexes (e.g., toeholds and toehold complements) if their structure free energies deviate from each other, or alternatively from a specified reference free energy.

Let S denote the user-specified set of soft constraints for a design problem.

### Constrained Multitube Design Problem

To design a set of sequences, Φ_Ψ_, for a multitube ensemble, Ω, subject to user-specified hard constraints R and soft constraints S , the constrained multitube design problem is

35 $$\min_{\phi_{\Psi }}\left[M_{W}+\sum_{k\in S}w_{k}\;f_{k}\left(\phi_{\Psi }\right)\right],\mathrm{subject}\;\mathrm{to}\;R$$

where $M_{W}$ is the multitube ensemble defect including user-specified defect weights W . The sequence design algorithm seeks to iteratively reduce the *augmented objective function* (weighted ensemble defect plus weighted soft constraints) below the *stop condition*

36 $$\left[M_{W}+\sum_{k\in S}w_{k}\;f_{k}\left(\phi_{\Psi }\right)\right]\leq f_{\mathrm{stop}}$$

for user-specified *f*_stop_ ∈ (0, 1) while satisfying the hard constraints in R .

## Analysis Page

The Analysis page of the NUPACK web app enables users to analyze the equilibrium concentration and base-pairing properties of a multitube ensemble containing one or more test tubes. Each test tube ensemble contains a user-specified set of strand species, each introduced at a user-specified concentration.

### Input

The Analysis Input page allows the user to specify the physical model and components for the multitube ensemble:

• *Material:* Specify a single-material job (RNA, DNA, or 2′OMe-RNA) or mixed-material job (RNA/DNA or RNA/2′OMe-RNA).
• *Temperature:* Specify the temperature in Celsius (or select “Melt” and specify a minimum temperature, increment, and maximum temperature to simulate the multitube ensemble for a range of temperatures).
• *Model options:* Optionally specify details of the physical model:
*− Parameters:* Select from available free energy parameter sets.
*− Ensemble:* Specify the coaxial and dangle stacking formulation.
*− Salts:* Specify salt concentrations.

For each test tube within the multitube ensemble, specify the following:

• *Tube name:* Specify the name of the test tube.
• *Strands:* Specify the name, sequence, and concentration of each strand species.
• *Complexes:* Specify the complex species in the test tube in any of three ways:
  − *Max complex size:* Automatically generate all complexes up to a specified maximum number of strands (default: 1).
  − *Include complex:* explicitly specify complexes to include in the test tube ensemble (that would otherwise not be included based on the specified “Max complex size”).
  − *Exclude complex:* explicitly specify complexes to exclude from the test tube ensemble (that would otherwise be included based on the specified “Max complex size”).

### Computation

For each complex in the multitube ensemble, the partition function, equilibrium base-pairing probabilities, and minimum free energy (MFE) proxy structure are calculated using a unified dynamic programming framework.^5,9^ If the same strand species are present in more than one tube of a multitube ensemble, the algorithms achieve significant cost savings relative to analyzing each tube in the ensemble separately.^9^ For each test tube ensemble, the equilibrium complex concentrations are the solution to a strictly convex optimization problem (formulated in terms of calculated partition functions and user-specified strand concentrations), which is solved efficiently in the dual form.^5^ The equilibrium complex concentrations and base-pairing probabilities are then used to calculate the test tube fraction of bases unpaired and test tube ensemble pair fractions.^5^ In order to analyze the tube properties for a different set of strand concentrations, it is not necessary to rerun the dynamic programs to calculate partition functions, equilibrium base-pairing probabilities, and MFE proxy structures; only the convex optimization problem must be solved again, and this can be rapidly done from within the Results page.

### Results

The Analysis Results page summarizes the equilibrium concentration and base-pairing properties of each test tube in the multitube ensemble; use the dropdown to examine the results for a given tube.

• *Temperature slider:* For calculations that specified a temperature range, use the temperature slider to examine results over the range of simulated temperatures.
• *Melt profile:* For calculations that specified a temperature range, the melt profile depicts the equilibrium fraction of bases unpaired in the test tube as a function of temperature.
• *Equilibrium complex concentrations:* The bar graph depicts the equilibrium concentration of each complex that forms with appreciable concentration in the test tube (adjust the display filters to alter which complex concentrations are shown). Click any bar to display equilibrium base-pairing information for the corresponding complex:
*− MFE structure:* Depicts the MFE proxy structure for the complex. In the default view, each base is shaded with the probability that it adopts the depicted base-pairing state at equilibrium, revealing which portions of the structure usefully summarize equilibrium structural features of the complex ensemble.
*− Pair probabilities:* Depicts equilibrium base-pairing probabilities for the complex. By definition, these data are independent of concentration and of all other complexes in solution. The area and color of each dot scale with the equilibrium probability of the corresponding base pair. With this convention, the matrix is symmetric, as denoted by a diagonal line. In the column at right, the area and color of each dot scale with the equilibrium probability that the corresponding base is unpaired within the complex ensemble. Optional black circles depict each base pair or unpaired base in the MFE proxy structure.

• *Test tube ensemble pair fractions:* Depicts equilibrium base-pairing information for the test tube ensemble, taking into account the equilibrium concentration and base-pairing properties of each complex. The area and color of the dot at row *i* and column *j* scale with the equilibrium fraction of base *i* that is paired to base *j* in solution. With this convention, the matrix can be asymmetric. In the column at right, the area and color of the dot in row *i* scales with the equilibrium fraction of base *i* that is unpaired within the test tube ensemble.

Information can be exported to the Design or Utilities pages:

• *To Design:* For a given complex, export the MFE proxy structure to the Design page to redesign the sequence.
• *To Utilities:* For a given complex, export the MFE proxy structure and sequence information to the Utilities page to annotate publication quality graphics, or to do quick analysis or design calculations in the context of the complex ensemble.

For individual plots, download graphics for editing in vector graphics programs or download data for local plotting. Alternatively, all job data and plots can be downloaded as a single compressed file.

## Design Page

The Design page of the NUPACK web app allows users to perform sequence design over a multitube ensemble comprising one or more target test tubes. See Design Formulation for details on how to formulate a multitube design problem.

### Input

The Design Input page allows the user to specify the physical model and components for the multitube ensemble:

• *Material:* Specify a single-material job (RNA, DNA, or 2′OMe-RNA) or mixed-material job (RNA/DNA or RNA/2′OMe-RNA).
• *Temperature:* Specify the temperature in Celsius.
• *Trials:* Specify the number of independent design trials.
• *Model options:* Optionally specify details of the physical model:
  − *Parameters:* Select from available free energy pa- rameter sets.
  − *Ensemble:* Specify the coaxial and dangle stacking formulation.
  − *Salts:* Specify salt concentrations.

• *Algorithm settings:* Optionally specify algorithm settings:
  − *Stop condition:* Specify a stop condition in the range (0,1). The design algorithm will attempt to reduce the augmented objective function (weighted ensemble defect plus weighted soft constraints) below the stop condition while satisfying hard constraints.
  − *Max design time:* Specify the maximum design time.
  − *Random seed:* Specify a non-zero integer for a re- producible design trial (default: 0; corresponding to a random trial).
  − *Wobble mutations:* Globally prohibit (default) or allow wobble mutations. Wobble mutations can also be allowed locally when specifying complementarity hard constraints.

Specify all components that appear in one or more target test tubes within the multitube ensemble:

• *Domains:* Specify sequence domains. A domain is a set of consecutive nucleotides that appear as a subsequence of one or more strands in the design, specified as a name and a sequence (specified 5′ to 3′ using degenerate nucleotide codes; see Table 1). Note that specification of a domain using degenerate nucleotide codes represents an implicit hard sequence constraint.
• *Strands:* Specify target strands. Each target strand is a single RNA, DNA, or 2′OMe-RNA molecule specified as a name and a sequence (specified 5′ to 3′ in terms of previously specified domains).
• *Target complexes:* Specify target complexes. Each target complex is an on-target and/or off-target complex specified as a name and an ordered list of strands (i.e., an ordering of strands around a circle in a polymer graph) and a complex name. If the complex is to be used as an on-target complex in at least one target test tube, it is specified with an on-target secondary structure (specified in dot-parens-plus, RLE dot-parents-plus, or DU+ notation); the target structure will be ignored in target test tubes where a complex appears as an off-target complex.

For each target test tube in the multitube ensemble, specify the following:

• *Target tube name:* Specify the name of the target test tube.
• *On-target complexes:* Specify a set of on-target complexes (from the previously specified set of target complexes that include a target secondary structure), each with a target concentration.
• *Off-target complexes:* Specify off-target complexes in any of three ways:
  − *Max complex size:* Automatically generate the set of all off-target complexes up to a specified maximum number of strands (default: 1).
  − *Include complex:* Explicitly specify off-target complexes to include in the test tube ensemble (that would otherwise not be included based on the specified “Max complex size”).
  − *Exclude complex:* Explicitly specify off-target complexes to exclude from the test tube ensemble (that would otherwise be included based on the specified “Max complex size”).

Note that any complex included as an on-target complex will not be included as an off-target complex. Note also that if an off-target is specified using a target complex for which a target structure has been specified, the target structure is ignored (by definition, there is no target structure for an off-target complex). Note further that used together, “Max complex size” and “Exclude complex” provide a powerful combination for specifying target test tubes. With “Max Complex Size”, it is possible to specify a large set of off-target complexes formed from a set of system components. With “Exclude complex”, it is further possible to remove from this large set all of the cognate products that should form between these system components (so they appear as neither on-targets nor off-targets in the tube ensemble). For example, with this approach, the reactive species in a global crosstalk tube can be forced to either perform no reaction (remaining as desired on-targets) or to undergo a crosstalk reaction (forming undesired off-targets), enabling minimization of global crosstalk during sequence optimization.

Optionally specify hard constraints, soft constraints, and/or defect weights for the multitube ensemble:

• *Hard constraints:* Specify hard constraints that prohibit sequences that violate the constraints, including match constraints, complementarity constraints, diversity constraints, similarity constraints, window constraints, library constraints, and pattern constraints.
• *Soft constraints:* Specify soft constraints that penalize (but do not prohibit) suboptimal sequences, including similarity constraints, pattern constraints, symmetry constraints, and energy match constraints.
• *Defect weights:* Specify defect weights to prioritize or deprioritize design quality for any combination of domain, strand, complex, or tube. Note that a defect weight can be specified as either an absolute weight or as a multiplier of existing weights.

See Design Formulation for details.

### Computation

The sequence design algorithm seeks to iteratively reduce the augmented objective function (weighted multitube ensemble defect plus weighted soft constraints) below a stop condition while satisfying the specified hard constraints. During sequence optimization, candidate mutations to a random initial sequence are efficiently evaluated over the multitube ensemble by estimating the multitube ensemble defect using test tube ensemble focusing, hierarchical ensemble decomposition, and conditional physical quantities calculated within subensembles.^6–8^ The progress page displays, for each independent design trial, the augmented objective function as a function of design time.

### Results

The following two plots summarize the design results for each independent design trial:

• *Augmented objective function:* This plot displays, for each independent design trial, the augmented objective function comprising:
  − The *weighted objective function*, incorporating any defect weights specified by the user. With the default value of unity for all weights, this reduces to the multitube ensemble defect.
  − The *weighted soft constraint* contribution for each soft constraint type specified by the user.
• *Multitube ensemble defect:* This plot displays, for each independent design trial, the multitube ensemble defect, representing the average equilibrium fraction of incorrectly paired nucleotides over the multitube ensemble. For each design trial, the defect contributions within the multitube ensemble come in two varieties:
  − The *structural defect* component quantifies the fraction of nucleotides that are in the incorrect base-pairing state within the correct complex.
  − The *concentration defect* component quantifies the fraction of nucleotides that are in an incorrect base-pairing state because they are not in the correct complex.

Click on the bar for any design trial to explore details for that design trial:

• *Tube defects:* This plot displays, for each target test tube, the *test tube ensemble defect*, representing the equilibrium concentration of incorrectly paired nucleotides over the ensemble of the test tube. For each target test tube, the defect contributions come in two varieties:
  − The *structural defect* component represents the equilibrium concentration of nucleotides that are in the incorrect base-pairing state within the correct complex.
  − The *concentration defect* component represents the equilibrium concentration of nucleotides that are in an incorrect base-pairing state because they are not in the correct complex.
• *Sequences:* Sequence design results are displayed for each sequence domain and each strand in the design ensemble.

Click on the bar for any tube to explore details for that tube:

• *On-target complex contribution to tube defect:* This plot displays, for each on-target complex, the contribution to the test tube ensemble defect, representing the equilibrium concentration of incorrectly paired nucleotides over the ensemble of the test tube. For each on-target complex, the defect contributions come in two varieties:
  − The *structural defect* component represents the equilibrium concentration of nucleotides that are in the incorrect base-pairing state within the ensemble of the complex.
  − The *concentration defect* component represents the equilibrium concentration of nucleotides that are in an incorrect base-pairing state because there is a deficiency in the concentration of the complex.
• *On-target complex defect:* This plot displays, for each on-target complex in the test tube, the *complex ensemble defect*, representing the equilibrium number of incorrectly paired nucleotides over the ensemble of the complex.
• *On-target complex concentration:* This plot displays, for each on-target complex in the test tube, the *equilibrium complex concentration* and the target concentration.
• *Off-target complex concentration:* This plot displays the equilibrium complex concentration for each off-target complex that forms appreciably in the test tube.

Click on the bar for any on-target complex to explore details for that complex:

• *Target structure:* Depicts the target secondary structure for the on-target complex. By default, each base is shaded with the probability that it adopts the depicted base-pairing state at equilibrium within the complex ensemble. Optionally, each base is shaded according to its identity.
• *Pair probabilities:* Depicts equilibrium base-pairing probabilities for the on-target complex. By definition, these data are independent of concentration and of all other complexes in solution. The area and color of each dot scale with the equilibrium probability of the corresponding base pair. With this convention, the matrix is symmetric, as denoted by a diagonal line. In the column at right, the area and color of each dot scale with the equilibrium probability that the corresponding base is unpaired within the complex ensemble. Optional black circles depict each base pair or unpaired base in the target structure.

Information can be exported to the Analysis or Utilities pages:

• *To Analysis:* For the multitube ensemble, a given target test tube, or a given on-target complex, export the designed sequences to the Analysis page for further equilibrium analysis.
• *To Utilities:* For a given on-target complex, export the target structure and designed sequences to the Utilities page to annotate publication quality graphics, or to do quick analysis or design calculations in the context of the complex ensemble.

For individual plots, download graphics for editing in vector graphics programs or download data for local plotting. Alternatively, all job data and plots can be downloaded as a single compressed file.

## Utilities Page

The Utilities page of the NUPACK web app allows users to analyze, design, or annotate the equilibrium properties of a complex. The page accepts as input either sequence information, structure information, or both, performing diverse functions based on the information provided, including:

• Evaluation and display of equilibrium base-pairing information for a specified secondary structure in the context of the complex to which it belongs.
• Automatic layout, rendering, and annotation of secondary structures specified in dot-parens-plus, RLE dot-parens-plus, or DU+ notation.
• Sequence analysis or design for a complex ensemble.

For individual plots, download graphics for editing in vector graphics programs or download data for local plotting. Alternatively, all job data and plots can be downloaded as a single compressed file.

Information can be exported to the Analysis or Design pages:

• *To Analysis:* For the specified complex, export the strand sequences to the Analysis page to analyze in the context of a test tube ensemble.
• *To Design:* For the specified complex, export the specified structure to the Design page to design the sequence in the context of a test tube ensemble, carrying along any specified sequence constraints.

Note that for a given complex ensemble:

• The Analysis page displays results through the lens of the MFE proxy structure.
• The Design page displays results through the lens of the target structure.
• The Utilities page displays results through the lens of a user-specified structure.

## Methods Summary

### NUPACK Web App Frontend

The NUPACK web app frontend is written in TypeScript using the React library for user interface logic and Semantic UI for user interface visuals. Bar graphs are generated using Plotly. Structure drawing and concentration calculations are written in C++17 and compiled to WebAssembly to run in the browser.

### NUPACK Web App Control Plane

The NUPACK web app control plane is written in Kotlin using PostgreSQL for metadata, AWS S3 for job storage, Redis for caching and communications, and Kubernetes for orchestration of worker containers and scaling clusters.

### NUPACK Web App Backend

The NUPACK 4 backend is written in C++17 using libsimdpp^42^ for SIMD operations, armadillo^43^ for linear algebra, Taskflow^44^ for task parallelization, and gecode^45^ for constraint solving.

### NUPACK Python Module

The NUPACK 4 Python module is written in C++17 with bindings to Python 3.8+. The numpy,^46^ scipy,^47^ and pandas^48^ packages are used to provide flexible user-friendly numerical interfaces.

## Resources

### NUPACK Documentation

Documentation is provided within the web app via the Overview page, the Definitions page, the expandable help text next to each item within the interface, and via the Intros and Demos for the Analysis, Design, and Utilities pages. Documentation for the NUPACK 4 Python module is provided via the NUPACK 4 User Guide (docs.nupack.org) including example jobs.

### NUPACK Software

NUPACK is a non-profit resource within the Beckman Institute at Caltech. Non-commercial academic users can subscribe to NUPACK to run jobs via the web app on the scalable hybrid cloud compute cluster subject to the NUPACK Terms, or to download the NUPACK 4 Python module subject to the NUPACK Software License Agreement (nupack.org). Commercial users can inquire about obtaining a commercial subscription and license by contacting info@nupack.org.

### NUPACK Technical Support

For technical support, feature requests, or bug reports, please contact support@nupack.org.
